# 2-[2-(4-Nitro­phenyl)hy­dra­zin­yl­idene]malononitrile

**DOI:** 10.1107/S1600536809053136

**Published:** 2009-12-19

**Authors:** Lu-Na Han, Min Zhang, Ran-Zhe Lu, Wen-Bin Wei, Hai-Bo Wang

**Affiliations:** a College of Science, Nanjing University of Technology, Xinmofan Road No. 5 Nanjing, Nanjing 210009, People’s Republic of China; b College of Food Science and Light Industry, Nanjing University of Technology, Xinmofan Road No. 5 Nanjing, Nanjing 210009, People’s Republic of China

## Abstract

The title compound, C_10_H_8_N_8_, is close to planar (r.m.s. deviation from the mean plane = 0.118 Å). In the crystal, inversion dimers linked by pairs of N—H⋯N hydrogen bonds generate *R*_2_^2^(12) loops.

## Related literature

For background to the use of the title compound as a dye, see: Tsai (2005 ▶). For reference structural data, see: Allen *et al.* (1987 ▶).
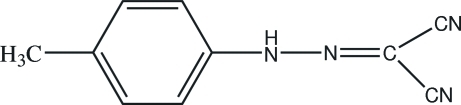


## Experimental

### 

#### Crystal data

C_10_H_8_N_4_
*M* *_r_* = 184.20
Monoclinic, 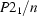

*a* = 11.961 (2) Å
*b* = 5.8310 (12) Å
*c* = 14.569 (3) Å
β = 110.98 (3)°
*V* = 948.7 (3) Å^3^
*Z* = 4
Mo *K*α radiation
μ = 0.08 mm^−1^
*T* = 293 K
0.30 × 0.20 × 0.10 mm

#### Data collection

Enraf–Nonius CAD-4 diffractometer
Absorption correction: ψ scan (North *et al.*, 1968 ▶) *T*_min_ = 0.975, *T*_max_ = 0.992
1797 measured reflections
1712 independent reflections
1191 reflections with *I* > 2σ(*I*)
*R*_int_ = 0.034
3 standard reflections every 200 reflections
intensity decay: 1%

#### Refinement

*R*[*F* ^2^ > 2σ(*F* ^2^)] = 0.059
*wR*(*F* ^2^) = 0.172
*S* = 1.01
1712 reflections
127 parameters
H-atom parameters constrained
Δρ_max_ = 0.25 e Å^−3^
Δρ_min_ = −0.25 e Å^−3^

### 

Data collection: *CAD-4 EXPRESS* (Enraf–Nonius, 1994 ▶); cell refinement: *CAD-4 EXPRESS*; data reduction: *XCAD4* (Harms & Wocadlo, 1995 ▶); program(s) used to solve structure: *SHELXS97* (Sheldrick, 2008 ▶); program(s) used to refine structure: *SHELXL97* (Sheldrick, 2008 ▶); molecular graphics: *SHELXTL* (Sheldrick, 2008 ▶); software used to prepare material for publication: *PLATON* (Spek, 2009 ▶).

## Supplementary Material

Crystal structure: contains datablocks global, I. DOI: 10.1107/S1600536809053136/hb5262sup1.cif

Structure factors: contains datablocks I. DOI: 10.1107/S1600536809053136/hb5262Isup2.hkl

Additional supplementary materials:  crystallographic information; 3D view; checkCIF report

## Figures and Tables

**Table 1:** Hydrogen-bond geometry (Å, °)

*D*—H⋯*A*	*D*—H	H⋯*A*	*D*⋯*A*	*D*—H⋯*A*
N4—H4*A*⋯N2^i^	0.86	2.36	3.174 (3)	157

Symmetry code: (i) 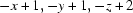.

**Fig. 1.**
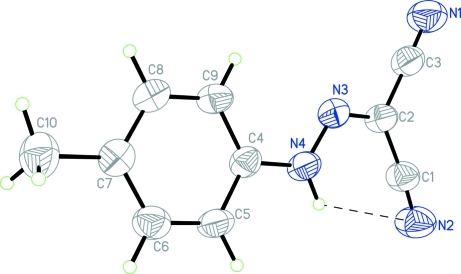
The molecular structure of (I) showing 50% displacement ellipsoids.

**Fig. 2.**
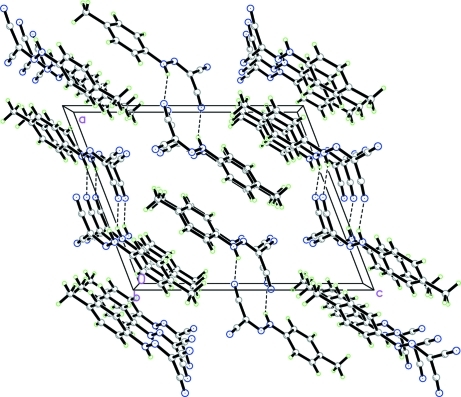
The packing for (I).

## supplementary crystallographic information

### Experimental

A hydrochloric acid solution (6 ml) of p-toluidine (1.07 g, 0.01 mol) and an aqueous solution (3 ml) of sodium nitrite (0.72 g, 0.0105 mol) were mixed and stirred at 273 K for 1h, followed by the addition of an aqueous solution (10 ml) of malononitrile (0.66 g, 0.01 mol) and further stirring at 273 K for 2 h. The resulting product was filtered and washed with water, dried, and recrystallized from ethanol to give the title compound as yellow crystals (yield; 78%, m.p. 409 K). Yellow blocks of (I) were obtained by slow evaporation of an ethyl acetate solution.

### Refinement

H atoms were positioned geometrically, with N-H = 0.86 Å (for NH) and C—H = 0.93, 0.95 and 0.96 Å for aromatic, methine and methyl H, respectively, and constrained to ride on their parent atoms, with U_iso_(H) = xU_eq_(C,N), where x = 1.5 for methyl H and x = 1.2 for all other H atoms.

### Crystal data

**Table:**

C_10_H_8_N_4_	*F*(000) = 384
*M**_r_* = 184.20	*D*_x_ = 1.289 Mg m^−^^3^
Monoclinic, *P*2_1_/*n*	Melting point: 409 K
Hall symbol: -P 2yn	Mo *K*α radiation, λ = 0.71073 Å
*a* = 11.961 (2) Å	Cell parameters from 25 reflections
*b* = 5.8310 (12) Å	θ = 10–13°
*c* = 14.569 (3) Å	µ = 0.08 mm^−^^1^
β = 110.98 (3)°	*T* = 293 K
*V* = 948.7 (3) Å^3^	Block, yellow
*Z* = 4	0.30 × 0.20 × 0.10 mm

### Data collection

**Table:**

Enraf–Nonius CAD-4 diffractometer	1191 reflections with *I* > 2σ(*I*)
Radiation source: fine-focus sealed tube	*R*_int_ = 0.034
graphite	θ_max_ = 25.3°, θ_min_ = 1.9°
ω/2θ scans	*h* = 0→14
Absorption correction: ψ scan (North *et al.*, 1968)	*k* = 0→7
*T*_min_ = 0.975, *T*_max_ = 0.992	*l* = −17→16
1797 measured reflections	3 standard reflections every 200 reflections
1712 independent reflections	intensity decay: 1%

### Refinement

**Table:**

Refinement on *F*^2^	Primary atom site location: structure-invariant direct methods
Least-squares matrix: full	Secondary atom site location: difference Fourier map
*R*[*F*^2^ > 2σ(*F*^2^)] = 0.059	Hydrogen site location: inferred from neighbouring sites
*wR*(*F*^2^) = 0.172	H-atom parameters constrained
*S* = 1.01	*w* = 1/[σ^2^(*F*_o_^2^) + (0.1*P*)^2^ + 0.170*P*] where *P* = (*F*_o_^2^ + 2*F*_c_^2^)/3
1712 reflections	(Δ/σ)_max_ < 0.001
127 parameters	Δρ_max_ = 0.25 e Å^−^^3^
0 restraints	Δρ_min_ = −0.25 e Å^−^^3^

### Special details

**Table:**

Geometry. All esds (except the esd in the dihedral angle between two l.s. planes) are estimated using the full covariance matrix. The cell esds are taken into account individually in the estimation of esds in distances, angles and torsion angles; correlations between esds in cell parameters are only used when they are defined by crystal symmetry. An approximate (isotropic) treatment of cell esds is used for estimating esds involving l.s. planes.
Refinement. Refinement of F^2^ against ALL reflections. The weighted R-factor wR and goodness of fit S are based on F^2^, conventional R-factors R are based on F, with F set to zero for negative F^2^. The threshold expression of F^2^ > 2sigma(F^2^) is used only for calculating R-factors(gt) etc. and is not relevant to the choice of reflections for refinement. R-factors based on F^2^ are statistically about twice as large as those based on F, and R- factors based on ALL data will be even larger.

### Fractional atomic coordinates and isotropic or equivalent isotropic displacement parameters (Å^2^)

**Table:**

	*x*	*y*	*z*	*U*_iso_*/*U*_eq_	
C1	0.2746 (2)	−0.1077 (5)	0.8662 (2)	0.0513 (6)	
N1	0.2481 (2)	−0.2797 (4)	0.8277 (2)	0.0739 (8)	
N2	0.5027 (2)	0.3075 (4)	0.92534 (19)	0.0692 (7)	
C2	0.4155 (2)	0.2173 (4)	0.91795 (18)	0.0489 (6)	
N3	0.24161 (17)	0.1817 (3)	0.96436 (14)	0.0445 (5)	
C3	0.3086 (2)	0.1060 (4)	0.91713 (17)	0.0448 (6)	
N4	0.26745 (16)	0.3729 (3)	1.01369 (14)	0.0445 (5)	
H4A	0.3268	0.4542	1.0123	0.053*	
C4	0.19927 (19)	0.4484 (4)	1.06931 (16)	0.0406 (6)	
C5	0.1092 (2)	0.3138 (4)	1.08016 (17)	0.0455 (6)	
H5A	0.0907	0.1720	1.0492	0.055*	
C6	0.0477 (2)	0.3931 (4)	1.13736 (18)	0.0486 (6)	
H6A	−0.0128	0.3027	1.1445	0.058*	
C7	0.0728 (2)	0.6037 (4)	1.18486 (17)	0.0461 (6)	
C8	0.1624 (2)	0.7356 (4)	1.17157 (18)	0.0492 (6)	
H8A	0.1806	0.8781	1.2019	0.059*	
C9	0.2249 (2)	0.6599 (4)	1.11451 (18)	0.0468 (6)	
H9A	0.2845	0.7512	1.1064	0.056*	
C10	0.0070 (2)	0.6860 (5)	1.2494 (2)	0.0630 (8)	
H10A	−0.0512	0.5734	1.2502	0.094*	
H10B	−0.0327	0.8281	1.2241	0.094*	
H10C	0.0629	0.7091	1.3150	0.094*	

### Atomic displacement parameters (Å^2^)

**Table:**

	*U*^11^	*U*^22^	*U*^33^	*U*^12^	*U*^13^	*U*^23^
C1	0.0532 (14)	0.0468 (15)	0.0609 (15)	−0.0056 (12)	0.0288 (12)	−0.0017 (13)
N1	0.0860 (18)	0.0582 (16)	0.0907 (19)	−0.0204 (13)	0.0476 (15)	−0.0187 (14)
N2	0.0589 (14)	0.0656 (16)	0.0930 (18)	−0.0156 (13)	0.0393 (13)	−0.0197 (14)
C2	0.0506 (15)	0.0408 (14)	0.0592 (15)	−0.0008 (12)	0.0244 (12)	−0.0060 (12)
N3	0.0498 (11)	0.0389 (12)	0.0464 (11)	−0.0051 (9)	0.0194 (9)	0.0010 (9)
C3	0.0432 (12)	0.0398 (13)	0.0539 (14)	−0.0042 (11)	0.0205 (11)	−0.0008 (11)
N4	0.0467 (11)	0.0377 (11)	0.0545 (12)	−0.0055 (9)	0.0246 (9)	0.0002 (9)
C4	0.0446 (12)	0.0355 (12)	0.0435 (12)	0.0002 (10)	0.0180 (10)	0.0053 (10)
C5	0.0470 (13)	0.0356 (13)	0.0546 (14)	−0.0059 (10)	0.0190 (11)	−0.0001 (11)
C6	0.0451 (13)	0.0475 (15)	0.0563 (14)	−0.0072 (11)	0.0219 (11)	0.0033 (12)
C7	0.0430 (13)	0.0475 (15)	0.0468 (13)	0.0037 (11)	0.0149 (10)	0.0057 (11)
C8	0.0549 (14)	0.0373 (13)	0.0556 (14)	−0.0026 (11)	0.0200 (12)	−0.0017 (11)
C9	0.0495 (13)	0.0367 (13)	0.0578 (14)	−0.0104 (11)	0.0236 (11)	−0.0005 (11)
C10	0.0573 (16)	0.0739 (19)	0.0632 (16)	0.0063 (14)	0.0281 (13)	−0.0021 (14)

### Geometric parameters (Å, °)

**Table:**

C1—N1	1.137 (3)	C5—H5A	0.9300
C1—C3	1.433 (3)	C6—C7	1.389 (3)
N2—C2	1.138 (3)	C6—H6A	0.9300
C2—C3	1.430 (3)	C7—C8	1.388 (3)
N3—N4	1.302 (3)	C7—C10	1.504 (3)
N3—C3	1.305 (3)	C8—C9	1.375 (3)
N4—C4	1.410 (3)	C8—H8A	0.9300
N4—H4A	0.8600	C9—H9A	0.9300
C4—C9	1.380 (3)	C10—H10A	0.9600
C4—C5	1.387 (3)	C10—H10B	0.9600
C5—C6	1.375 (3)	C10—H10C	0.9600
			
N1—C1—C3	178.5 (3)	C7—C6—H6A	118.9
N2—C2—C3	175.3 (3)	C8—C7—C6	117.4 (2)
N4—N3—C3	120.74 (19)	C8—C7—C10	120.9 (2)
N3—C3—C2	123.9 (2)	C6—C7—C10	121.7 (2)
N3—C3—C1	117.0 (2)	C9—C8—C7	121.4 (2)
C2—C3—C1	119.1 (2)	C9—C8—H8A	119.3
N3—N4—C4	120.83 (19)	C7—C8—H8A	119.3
N3—N4—H4A	119.6	C8—C9—C4	119.9 (2)
C4—N4—H4A	119.6	C8—C9—H9A	120.0
C9—C4—C5	120.0 (2)	C4—C9—H9A	120.0
C9—C4—N4	118.5 (2)	C7—C10—H10A	109.5
C5—C4—N4	121.4 (2)	C7—C10—H10B	109.5
C6—C5—C4	119.1 (2)	H10A—C10—H10B	109.5
C6—C5—H5A	120.5	C7—C10—H10C	109.5
C4—C5—H5A	120.5	H10A—C10—H10C	109.5
C5—C6—C7	122.1 (2)	H10B—C10—H10C	109.5
C5—C6—H6A	118.9		
			
N4—N3—C3—C2	2.4 (4)	N4—C4—C5—C6	178.3 (2)
N4—N3—C3—C1	179.0 (2)	C4—C5—C6—C7	−0.1 (4)
N2—C2—C3—N3	45 (3)	C5—C6—C7—C8	0.9 (4)
N2—C2—C3—C1	−131 (3)	C5—C6—C7—C10	−178.4 (2)
N1—C1—C3—N3	−65 (10)	C6—C7—C8—C9	−0.7 (4)
N1—C1—C3—C2	112 (10)	C10—C7—C8—C9	178.6 (2)
C3—N3—N4—C4	−176.4 (2)	C7—C8—C9—C4	−0.2 (4)
N3—N4—C4—C9	−175.4 (2)	C5—C4—C9—C8	1.0 (3)
N3—N4—C4—C5	5.4 (3)	N4—C4—C9—C8	−178.1 (2)
C9—C4—C5—C6	−0.9 (3)		

### Hydrogen-bond geometry (Å, °)

**Table:**

*D*—H···*A*	*D*—H	H···*A*	*D*···*A*	*D*—H···*A*
N4—H4A···N2^i^	0.86	2.36	3.174 (3)	157

Symmetry codes: (i) −*x*+1, −*y*+1, −*z*+2.
